# Bacterial Cyclodipeptides Inhibit Invasiveness and Metastasis Progression in the Triple-Negative Breast Cancer MDA-MB-231 Mouse Model

**DOI:** 10.3390/molecules31030543

**Published:** 2026-02-04

**Authors:** Mayra Xochitl Durán-Maldonado, Ximena Hernández-Ramos, Marlene Estefania Campos-Morales, Lorena Martínez-Alcantar, Laura Hernández-Padilla, Jesús Campos-García

**Affiliations:** 1 Instituto de Investigaciones Químico Biológicas, Universidad Michoacana de San Nicolás de Hidalgo, Morelia 58030, Michoacan, Mexico; 1028165h@umich.mx (M.X.D.-M.); 1590734d@umich.mx (X.H.-R.); 1435830h@umich.mx (M.E.C.-M.); lorena.martinez@umich.mx (L.M.-A.); 0483419a@umich.mx (L.H.-P.); 2 Secretaría de Ciencia, Humanidades, Tecnología e Innovación (SECIHTI), Investigadora por México, Universidad Michoacana de San Nicolás de Hidalgo, Morelia 58030, Michoacan, Mexico

**Keywords:** cyclodipeptides, anti-metastatic, triple-negative breast cancer

## Abstract

Triple-negative breast cancer (TNBC) is a highly aggressive subtype linked to a high rate of metastasis and low survival rates worldwide. Bacterial cyclodipeptides (CDPs) demonstrate anticancer properties by targeting multiple signaling pathways. The impact of CDPs on TNBC metastasis was evaluated both in vitro and in advanced-stage tumors in immunosuppressed female mice. CDPs significantly decreased the migratory and invasive capabilities of the MDA-MB-231 cell line, outperforming methotrexate (MTX). This effect was associated with the inhibition of Akt/mTOR/S6K phosphorylation, as well as Gab1, Vimentin, and FOXO1. Mice bearing MDA-MB-231 xenografts treated with CDPs alone or in combination with MTX showed near-complete suppression of primary tumors and metastatic sites in organs; notably, the combined treatment displayed a synergistic effect. Consequently, key proteins involved in tumor progression and metastasis, including p-Akt, p-Gab1, and FOXO1, were markedly inhibited in tumors from CDP-treated mice. Additionally, genes related to EMT, invasiveness, and immune modulation—including PTEN, SNAIL, CXCL1, BRCA1, GADD45A, and PD-L1—were dysregulated in the livers of TNBC-bearing mice; however, CDP treatment restored their expression more effectively than MTX. These findings suggest that the anti-metastatic effects of CDPs in the TNBC xenograft model involve modulation of the Akt/mTOR/S6K pathway, EMT, invasiveness, and immune modulation, highlighting their potential for further preclinical development.

## 1. Introduction

Breast cancer is one of the leading causes of cancer-related mortality among women worldwide and represents a clinically heterogeneous disease. Approximately 10–15% of patients develop highly aggressive tumors with an increased propensity for metastasis [1]. Triple-negative breast cancer (TNBC) comprises a heterogeneous group of tumors with distinct histological, genomic, and immunological characteristics and is defined by the absence of estrogen and progesterone hormone receptors, as well as human epidermal growth factor receptor 2 (HER2). Several risk factors, including obesity, are associated with poor prognosis, as the interaction between adipocytes and tumor cells creates a permissive tumor microenvironment that promotes cancer cell proliferation, invasion, metastatic dissemination, and resistance to cell death [2].

In the cancer context, liver metastasis represents one of the most challenging complications in TNBC and is associated with high rates of morbidity and mortality. The liver is among the most frequent sites of breast cancer dissemination and constitutes the initial metastatic organ in approximately 30% of individuals with metastatic breast cancer. The development of liver metastases is associated with an unfavorable prognosis and reduced overall survival, even in the context of advances in diagnostic and therapeutic strategies achieved in recent decades. Notably, TNBC patients with liver involvement exhibit a poorer clinical outcome compared with those affected by other breast cancer subtypes. Consequently, the identification of novel therapeutic strategies capable of improving outcomes in patients with metastatic breast cancer remains a critical unmet clinical need.

The FDA has approved several drugs with therapeutic uses targeting members of the protein kinase superfamily (PKs) associated with various diseases. Because the ATP-binding site is conserved across the human kinome, ATP-mimicking compounds often interact with multiple kinases. Drugs such as Abemaciclib, Palbociclib, Ribociclib, and Trilaciclib target the CDK4/6 proteins at the ATP-binding site; therefore, kinase-like proteins such as CDK4/6, AKT, HER2, ErbB1/2/4, and FKBP12/mTOR are considered therapeutic targets for breast cancer treatment [3]. In contrast, inhibitors designed for a single target, like the kinase inhibitor Capivasertib, which targets the AKT oncogene—a member of the PI3K/Akt/mTOR signaling pathway—have become key in treating cancer, diabetes, obesity, neurodegenerative diseases, aging, and autoimmunity [3]. Among the primary treatment options for metastatic breast cancer are taxanes, anthracyclines, methotrexate (MTX), carboplatin, immune checkpoint inhibitors targeting PD-1 or PD-L1, PARP inhibitors, and combinations of these agents with immunotherapy, radiotherapy, or surgery [4]. However, a significant number of patients respond poorly to these treatments, leading to low survival rates and a reduced quality of life. As a result, the pursuit of new therapeutic strategies that can improve the prognosis for patients with metastatic breast cancer remains a vital priority.

Cyclodipeptides (CDPs) are a class of molecules of the diketopiperazine family, produced by a wide range of organisms that exhibit notable biological activities, including cytotoxic effects against multiple cancer cell lines [5,6,7,8]. Previously, we reported that CDPs, such as cyclo(L-Pro-L-Tyr), cyclo(L-Pro-L-Val), cyclo(L-Pro-L-Leu), and cyclo(L-Pro-L-Phe), isolated from the *Pseudomonas aeruginosa* PAO1 bacterium, induce apoptosis in cervical, colon, leukemia, and melanoma cancer cells. In HeLa cells, CDPs inhibit cell proliferation, including arresting cells at the G0–G1 stage, and induce apoptosis via an intrinsic pathway that relies on caspase-9 and -3 activation. Mechanistically, the antiproliferative effects of CDPs involve modulation of multiple signaling networks associated with tumor growth, survival, and plasticity, including the PI3K/Akt/mTOR, Ras/Raf/MEK/ERK1/2, PI3K/JNK/PKA, p27Kip1/CDK1/survivin, MAPK, HIF-1, Wnt/β-catenin, HSP27, epithelial–mesenchymal transition (EMT), and cancer stem cell (CSCs) pathways [3,6,7]. Transcriptomic profiling of HeLa cells treated with CDPs revealed 151 differentially expressed genes distributed across 15 major biological processes, including regulation, signaling, development, locomotion, adhesion, metabolism, growth, proliferation, and immune response. Pathway enrichment analysis suggested that CDPs modulate multiple signaling networks, encompassing the insulin/IGF/MAP kinase cascade, P38/MAPK signaling, interleukin-mediated pathways, epidermal growth factor receptor (EGFR), PI3K signaling, platelet-derived growth factor (PDGF), Notch, cadherin, p53, Hedgehog, transforming growth factor-β (TGF-β), fibroblast growth factor (FGF), FAS-mediated signaling, angiogenesis, cholesterol biosynthesis, and pathways associated with inflammation, oxidative stress responses, apoptosis, cytoskeletal organization, and T- and B-cell activation [9]. In a murine melanoma model, CDP treatment reduced tumor growth by decreasing the expression of proteins that mediate pathways involved in energy metabolism, lipid synthesis, EMT, invasion, and metastasis [10]. In this context, cyclic peptides have emerged as promising therapeutic agents for kinase inhibition, as dysregulated kinases play central roles in cancer cell survival, proliferation, metastasis, and drug resistance. Given this background, we sought to determine whether bacterial CDPs have a promising effect on metastatic breast cancer, including advanced stages of tumorigenesis using a xenograft model of triple-negative breast cancer established with MDA-MB-231 (ER-, PR-, HER2-) cells.

## 2. Results

### 2.1. Bacterial CDPs Reduce Migration and Invasiveness in the MDA-MB-231 Breast Cancer Cell Line

Previously, the cytotoxic and apoptotic properties of the CDPs have been demonstrated in HeLa and CaCo-2 [8] and murine melanoma lines [10]; however, their anti-metastatic potential remains unknown. Therefore, the MDA-MB-231 cell line was used as a model to study triple-negative breast cancer and evaluate the anti-metastatic properties of CDPs.

**Figure 1:**
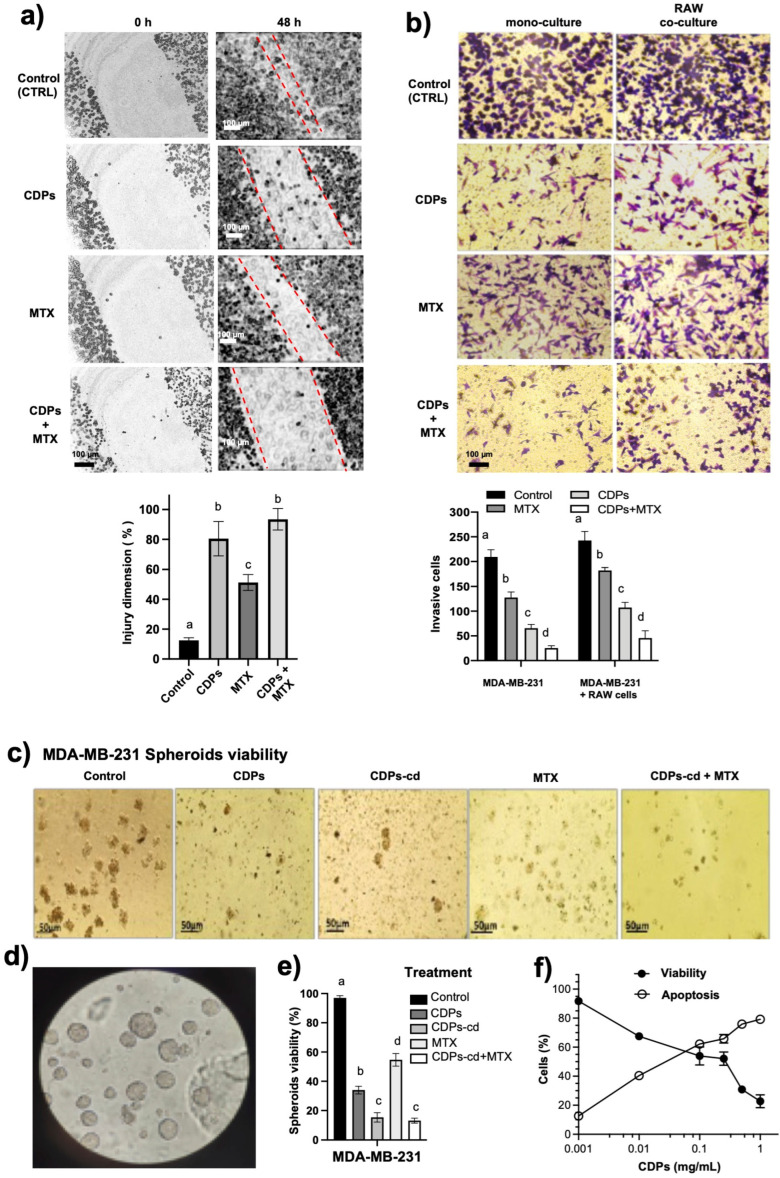
Effects of bacterial CDPs on the migratory and invasive properties of MDA-MB-231 breast cancer cells. (**a**) Representative photographs of wound closure migration assays taken at zero h and 48 h in MDA-MB-231 cell cultures treated with the saline solution (CTRL), CDPs (0.01 mg/mL), MTX (0.05 mg/mL), or combined CDP + MTX treatment (0.01 mg/mL + 0.05 mg/mL, respectively) (**b**) Representative photographs of the cancer cells that managed to invade the Matrigel-covered membrane, stained with crystal violet. The monoculture conditions and co-culture with RAW 264.7 macrophages are shown. Treatment were CDPs (0.01 mg/mL), MTX (0.05 mg/mL), or combined CDP + MTX treatment (0.01 mg/mL + 0.05 mg/mL, respectively) by 24 h. Below, the quantification of the wound dimension expressed in percentage (**a**) and the number of invasive cells (**b**) is done using ImageJ 1.53k Software. (**c**,**d**) Representative photographs of multicellular spheroids of the MDA-MB-231 line. (**e**) Cell viability of the spheroids treated with CDPs (0.1 mg/mL) plus MTX (0.05 mg/mL) determined by the MTT method after 24 h of incubation. (**f**) Cell viability and apoptosis induction of the MDA-MB-231 cell cultures submitted to CDP treatments for 4 h, determined by FACS. Bars represent means ± SE, n = 3. Statistical analysis was performed using one-way ANOVA followed by Tukey’s post hoc test. SE values are shown as bars; groups sharing the same lowercase letter are not significantly different from each other, while groups with different letters indicate statistically significant differences (*p* < 0.05). CDPs, cyclodipeptides; MTX, methotrexate; CDPs-cd, CDPs dissolved in β-cyclodextrins; CDPs-cd + MTX, combined treatment.

In wound-healing assays, untreated MDA-MB-231 monolayers recovered about >90% of the original wound area after 48 h. Meanwhile, the chemotherapeutic compound methotrexate (MTX; 0.05 mg/mL), used as an anti-migration control, resulted in about 40–50% of the wound remaining unclosed during the same period. In contrast, CDP treatment (0.01 mg/mL), whether alone or combined with MTX, significantly inhibited cell migration, leaving approximately 80% and 90% of the wound area unclosed after 48 h, respectively (Figure 1a).

Furthermore, since chemokine-mediated macrophage signaling enhances tumor invasiveness, the invasive ability of MDA-MB-231 cells was assessed using a transwell assay. Co-culturing with RAW macrophages increased the number of invading cancer cells, confirming the pro-invasive role of the macrophage microenvironment (Figure 1b). CDP treatment significantly reduced invasion, lowering the number of invading cells by approximately 75% in monoculture and about 60% in macrophage co-culture conditions. In comparison, invasion under MTX (0.05 mg/mL) treatment was less suppressed than with CDPs alone (0.01 mg/mL), but was most effectively inhibited when CDPs were combined with MTX, resulting in up to a 90% reduction in invasiveness (Figure 1b).

Additionally, spheroids are cellular aggregates that represent three-dimensional cultures of cancer cells and serve as models for tumor development. Therefore, the effect of CDPs on the viability and integrity of spheroids from the MDA-MB-231 cell line was assessed (Figure 1c,d). A cyclodextrin suspension, consistent with previous in vivo findings, was used as the delivery vehicle [10]. The results indicated a significant decrease in both the number and size of spheroids in the MDA-MB-231 cell line when treated with CDPs alone (0.1 mg/mL) or combined with cyclodextrins (CDPs-cd). The CDPs at 0.1 mg/mL showed a strong ability to disrupt spheroid formation, similar to MTX (0.05 mg/mL). Notably, the most effective outcome was observed with the combination of CDPs and the anti-neoplastic drug MTX (CDPs-cd + MTX) (Figure 1c–e). Spheroid viability and apoptosis induction were further assessed using MTT assays and flow cytometry, respectively. These analyses identified a lethal dose (LD50) of 0.25 mg/mL and an effective dose for apoptosis induction (ED50) of 0.02 mg/mL for CDPs after 4 h of treatment (Figure 1e,f).

### 2.2. Effect of Bacterial CDPs on the Akt/mTOR/S6K Signaling Pathway in the MDA-MB-231 Cell Line

**Figure 2:**
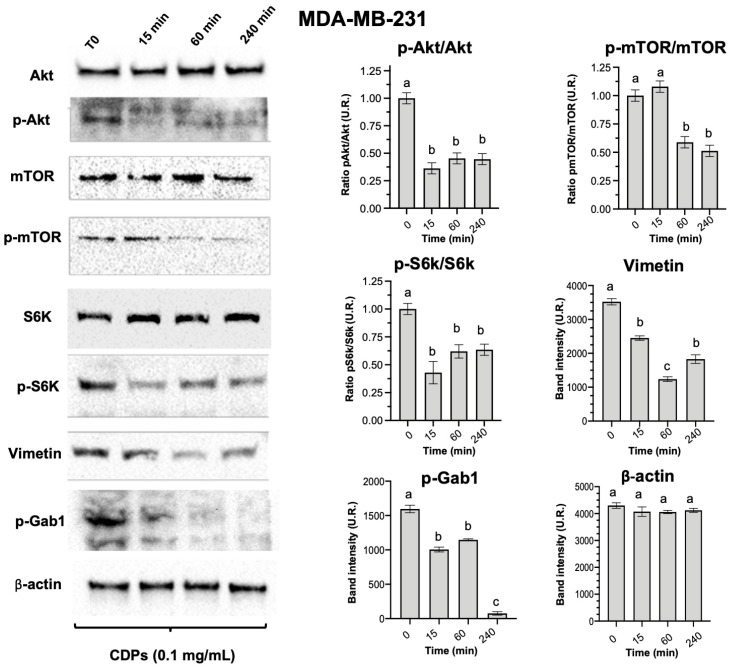
Effect of bacterial CDPs on proteins involved in proliferation, invasion, and metastasis in MDA-MB-231 triple-negative breast cancer cells. Protein extracts obtained from cell cultures treated with CDPs (0.1 mg/mL) over time were subjected to immunodetection. Protein expression for the total Akt, phosphorylated Akt, total mTOR, phosphorylated mTOR, total S6K, phosphorylated S6K, Vimentin, phosphorylated Gab1, and β-actin is shown. Densitometric analyses of the protein immunodetection results are presented on the right. The bars represent the means ± SE; n = 3 per group. Statistical analysis was performed using one-way ANOVA, followed by Tukey’s post hoc test; SE values are shown as bars; groups sharing the same lowercase letter are not significantly different from each other, while groups with different letters indicate statistically significant differences (*p* < 0.05).

The signaling pathways related to cell survival and growth were examined to explore the anti-neoplastic effects of bacterial CDPs in the MDA-MB-231 cell line. Results showed that CDP treatment (0.1 mg/mL) did not change the overall levels of Akt, mTOR, and S6K proteins and had no time-dependent effects on their expression; however, their phosphorylation states—p-Akt, p-mTOR, and p-S6K—were significantly decreased over time after CDP treatment (Figure 2). Therefore, the ratio of phosphorylated forms to total protein was significantly reduced. Additionally, phosphorylation of p-Gab1 and the expression of Vimentin were notably decreased in MDA-MB-231 cells following CDP treatment (Figure 2).

### 2.3. Effect of Bacterial CDPs in a Breast Cancer Mouse Model Through Implantation of the MDA-MB-231 Cell Line

**Figure 3:**
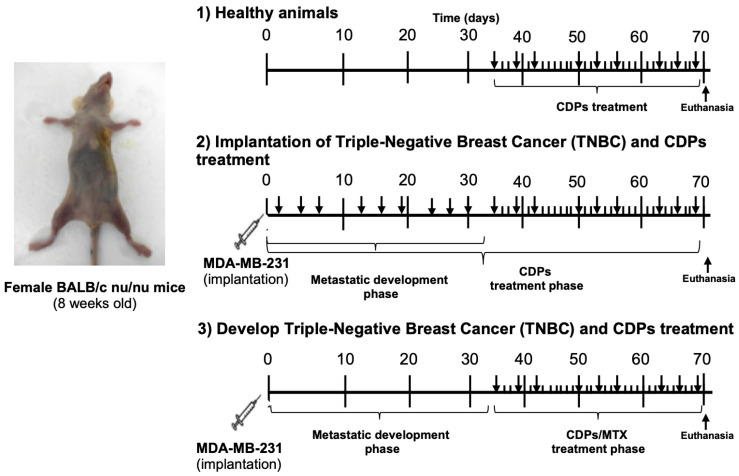
Therapeutic procedures in the MDA-MB-231 immunosuppressed mouse model of triple-negative breast cancer treated with bacterial CDPs. Female BALB/c nu/nu mice (8 weeks old) were used to establish an orthotopic triple negative breast cancer xenograft model. Three experimental treatment protocols were tested: (1) Healthy animals were treated or not with CDPs at 35 days after the start of the procedure (indicated by arrows), followed by nine doses of CDPs (0.25 mg/kg) over 35 days. (2) Mice were orthotopically inoculated with MDA-MB-231 cells and simultaneously treated with CDPs, followed by nine doses of CDPs (0.25 mg/kg) during 35 days (indicated by arrows). Treatment was then continued for an additional 35 days in the corresponding groups. (3) The third treatment protocol, mammary tumors were allowed to develop for 35 days following MDA-MB-231 cell implantation. After that, nine doses of CDPs (0.25 mg/kg), MTX (0.05 mg/kg), or both CDPs and MTX were administered during 35 days (arrows). Finally, at 70 days from the start of treatment, all animals were ethically euthanized.

An orthotopic TNBC xenograft model with metastatic dissemination was established by implantation of MDA-MB-231 cells into the mammary fat pad of immunosuppressed female mice, achieved 100% mammary tumor formation. Healthy mouse groups (procedure 1, Figure 3) and two treatment conditions were tested. In the second treatment regimen (procedure 2, Figure 3), mice were inoculated with MDA-MB-231 cells and treated simultaneously with CDPs, followed by a total of nine doses of CDPs (0.25 mg/kg) over a 35-day therapeutic period. In the third regimen (procedure 3, Figure 3), tumors were permitted to develop for 35 days after implanting MDA-MB-231 cells; then, treatment with CDPs or MTX (0.05 mg/kg) was started and continued for another 35 days.

Results of body weight monitoring showed that weight gain was similar across all mouse groups, except for the TNBC groups treated with CDPs (TNBC + CDPs) or with the combination of CDPs and MTX (TNBC + CDPs + MTX), which exhibited lower body weight gain slopes compared to untreated TNBC mice (Figure S1).

**Figure 4:**
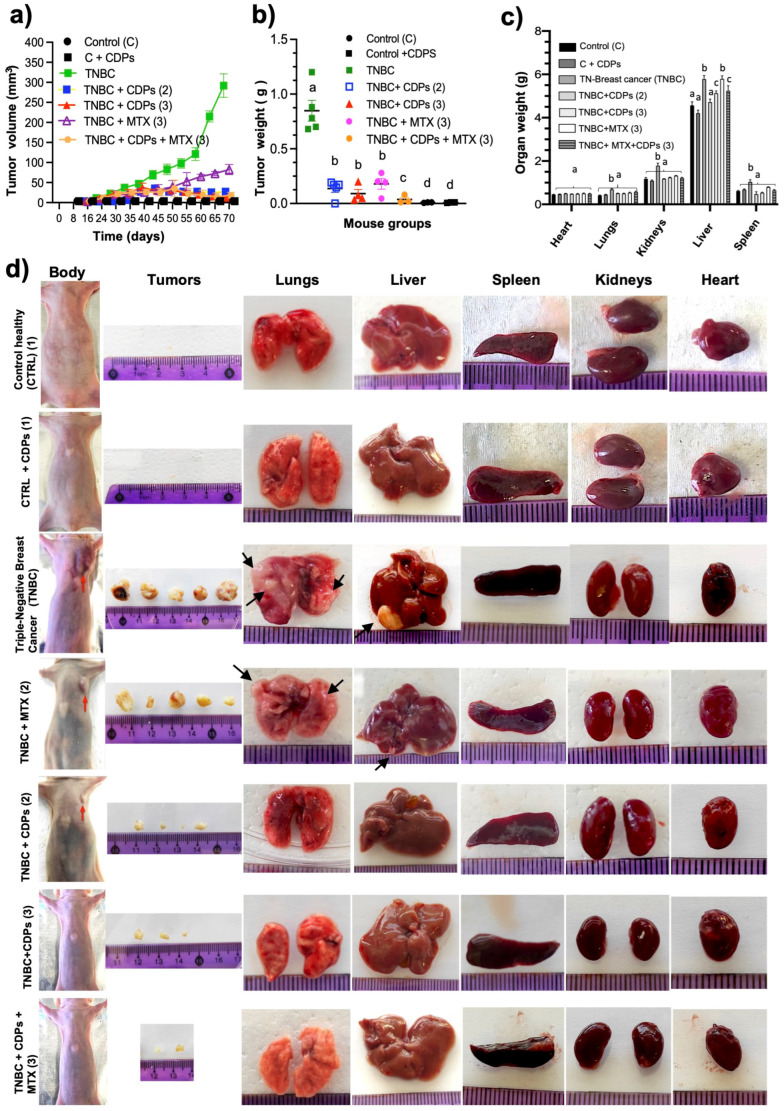
Antitumor effects of bacterial CDPs in an MDA-MB-231 xenograft mouse model of triple-negative breast cancer. (**a**) Tumor volume expressed in mm^3^ was monitored during the 70 days of the therapeutic procedure. (**b**) Tumor weight expressed in grams. (**c**) Weight of organs in grams. (**d**) Representative photographs of animals, tumors, and organs from each mouse group are shown, n = 5 per group. Arrows indicate metastatic foci in organs. Mouse groups: (CTRL), control healthy animals without treatment (procedure 1); CTRL + CDPs, healthy animals administered with CDPs (procedure 1); TNBC, animals that developed breast cancer without treatment (procedure 2); TNBC + CDPs (2), TNBC animals administered with CDPs (procedure 2); TNBC + CDPs (3), TNBC animals administered with CDPs (procedure 3); TNBC + MTX (3), TNBC animals administered with methotrexate (MTX) as procedure 3; TNBC + CDPs + MTX (3), TNBC animals co-administered with CDPs + MTX as procedure 3. Graphs show means ± SE; n = 5. Statistical analysis was performed using one-way ANOVA, followed by Tukey’s post hoc test. SE values are shown as bars; groups sharing the same lowercase letter are not significantly different from each other, while groups with different letters indicate statistically significant differences (*p* < 0.05).

The tumor volume was monitored in all mouse groups throughout the therapeutic experiment. On day 70, tumors in the untreated TNBC group reached an average volume of approximately 300 mm^3^ (Figure 4a). In contrast, TNBC mice treated with CDPs showed a marked reduction in tumor volume, either from the time of MDA-MB-231 cell implantation or after the tumor establishment phase (procedures 2 and 3, respectively), with a significant reduction to approximately 10 mm^3^ and 3 mm^3^, respectively. This indicates that both CDP treatment regimens were more effective than MTX, in which tumors reached an average volume of about 90 mm^3^ (Figure 4a).

After euthanasia, primary tumors and organs were dissected for detailed analysis (Figure 4b,c). Tumors from the untreated TNBC group weighed about 0.9 g on average. In contrast, the TNBC mice treated with CDPs showed significantly lower tumor weights, averaging around 0.15 g in the preventive treatment regimen (procedure 2) and approximately 0.05 g in the therapeutic regimen (procedure 3) (Figure 4b). Similarly, the MTX-treated group had a mean tumor weight of roughly 0.15 g. Notably, in the TNBC + CDPs and TNBC + CDPs + MTX groups, tumor weights were greatly reduced to about 0.05 g, and in 40–60% of these groups, no tumors were observed (Figure 4d).

In the orthotopic xenograft TNBC model, mice with mammary tumors that received no treatment (TNBC) showed a significant increase in the weights of the lungs, kidneys, liver, and spleen (Figure 4c,d), along with metastatic foci in the lungs and liver. In contrast, the groups treated with CDPs and the combination of CDPs plus MTX exhibited normalized organ weights and no visible metastatic foci (Figure 4c,d).

### 2.4. Effect of Bacterial CDPs on Biochemical and Hematological Parameters in the Breast Cancer Mouse Model with MDA-MB-231 Cell Line Implantation

**Figure 5:**
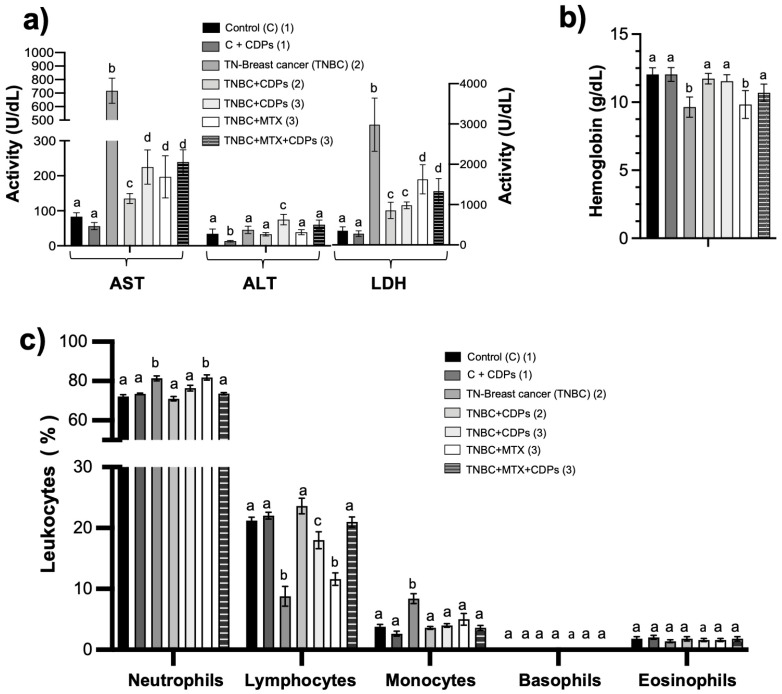
Hematological parameters in xenografted triple-negative breast cancer MDA-MB-231 cells in immunosuppressed mice treated with bacterial CDPs. (**a**) Measurement of enzyme activity of AST, ALT, and LDH in the serum of the mouse groups. (**b**) Hemoglobin levels. (**c**) Hematological profile of leukocyte counts. Group nomenclature is consistent with that described in Figure 4. Graphs display means ± SE; n = 5. Statistical analysis was conducted using one-way ANOVA, followed by Tukey’s post hoc test. SE values are shown as bars; groups sharing the same lowercase letter are not significantly different from each other, while groups with different letters indicate statistically significant differences (*p* < 0.05).

The enzymes alanine transaminase (ALT), aspartate transaminase (AST), and lactate dehydrogenase (LDH), used as markers of hepatocellular injury and systemic tissue damage, were measured in the blood serum of animals implanted with breast tumors. The untreated TNBC mice group showed a significant increase in AST and LDH enzyme activities compared with healthy controls (Figure 5a), which was reversed by treatment with CDPs, MTX, or their combination. Importantly, treatment with CDPs in the healthy control mice group (C + CDPs) did not affect AST or LDH levels, as seen in the control group.

The hematological analysis indicated that blood hemoglobin levels were significantly lower in mice with TNBC tumors and in the TNBC + MTX group; however, hemoglobin levels were restored in mice with tumors treated with CDPs, nearly reaching the levels of the healthy control group (Figure 5b). Additionally, leukocyte profiling revealed changes in neutrophil, lymphocyte, and monocyte counts in the TNBC and TNBC + MTX groups (Figure 5c). These changes were normalized in the groups treated with CDPs, which showed leukocyte distributions similar to healthy controls. Furthermore, healthy mice treated with CDPs did not exhibit any changes in these hematological parameters (Figure 5c).

### 2.5. Histological and Protein Expression in Tumors from the Xenografted TNBC MDA-MB-231 Line Treated with Bacterial CDPs

**Figure 6:**
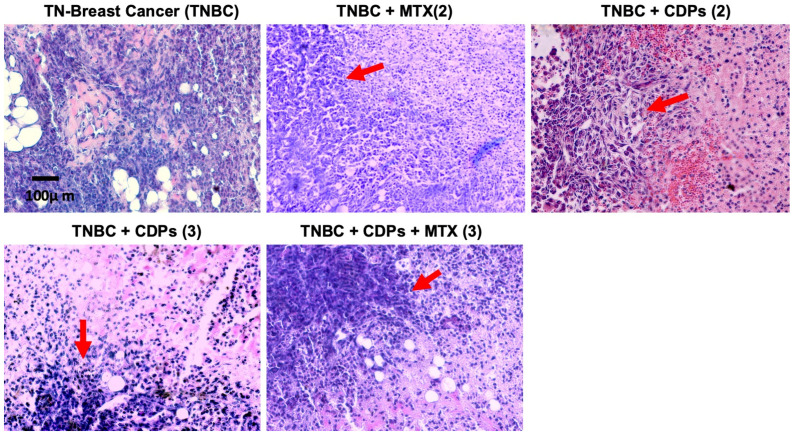
Histopathological features of tumors in an MDA-MB-231 xenograft mouse model of triple-negative breast cancer treated with bacterial CDPs. Female BALB/c nu/nu mice (8 weeks old) were subjected to antitumor treatments and analyzed at experimental endpoints corresponding to 35 or 70 days (n = 5 per group). Tumor sections were stained with hematoxylin and eosin (H&E) and examined by light microscopy. Representative images of tissues from the animal groups are shown. Regions of tumor necrosis and inflammatory cell infiltration are indicated by red arrows.

Histological analyses of tumors were conducted using hematoxylin and eosin (H&E) staining. Tumors from untreated TNBC mouse groups displayed nuclear hyperchromasia and infiltration of inflammatory cells (Figure 6). Notably, tumors from TNBC mouse groups treated with MTX or CDPs showed reduced nuclear hyperchromasia. In these treated groups, focal areas of necrosis and inflammatory cell infiltration were observed, indicating treatment-related histopathological changes (Figure 6).

**Figure 7:**
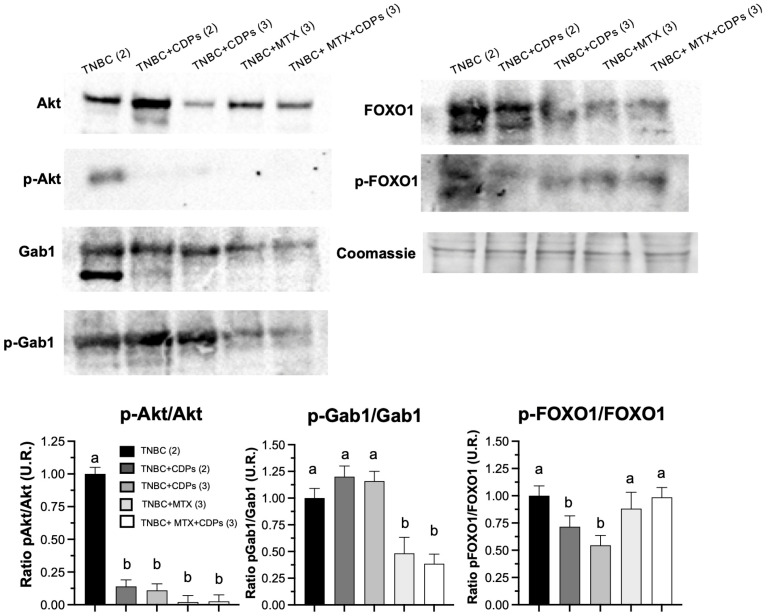
Effects of bacterial CDPs on signaling proteins in tumors grafted with MDA-MB-231 triple-negative breast cancer cells in immunosuppressed mice. Protein extracts from the tumors excised from the mouse groups (administered with CDPs, MTX, and co-administered CDPs + MTX) were subjected to immunodetection. Group nomenclature is consistent with that described in Figure 4. Representative immunoblots show total and phosphorylated forms of Akt, Gab1, and FOXO1. Densitometric analyses of the immunodetection results are presented below. The bars represent the means ± SE; n = 3 per group. Statistical analysis was conducted using one-way ANOVA, followed by Tukey’s post hoc test. SE values are shown as bars; groups sharing the same lowercase letter are not significantly different from each other, while groups with different letters indicate statistically significant differences (*p* < 0.05).

Protein immunodetection analysis was carried out on crude tumor extracts from the xenograft model. Results indicated that CDPs and MTX treatments significantly decreased Akt phosphorylation; notably, the ratio of the phosphorylated form to total Akt protein levels was greatly influenced by these treatments (Figure 7). The Gab1 protein expression ratio between the phosphorylated form and the total protein was significantly reduced in the TNBC + MTX(3) and TNBC + CDPs + MTX(3) groups. Similarly, the levels of FOXO1 and its phosphorylated form (p-FOXO1) were considerably lower in tumors from mice treated with CDPs, MTX, or both in combination. However, the ratio of phosphorylated p-FOXO1 to total protein was significantly lower in the TNBC + MTX(2) and TNBC + MTX(3) groups but was restored in the TNBC + MTX(3) and TNBC + CDPs + MTX(3) groups (Figure 7).

### 2.6. Histological of Organs from Xenografted TNBC MDA-MB-231 Mice Treated with Bacterial CDPs

**Figure 8:**
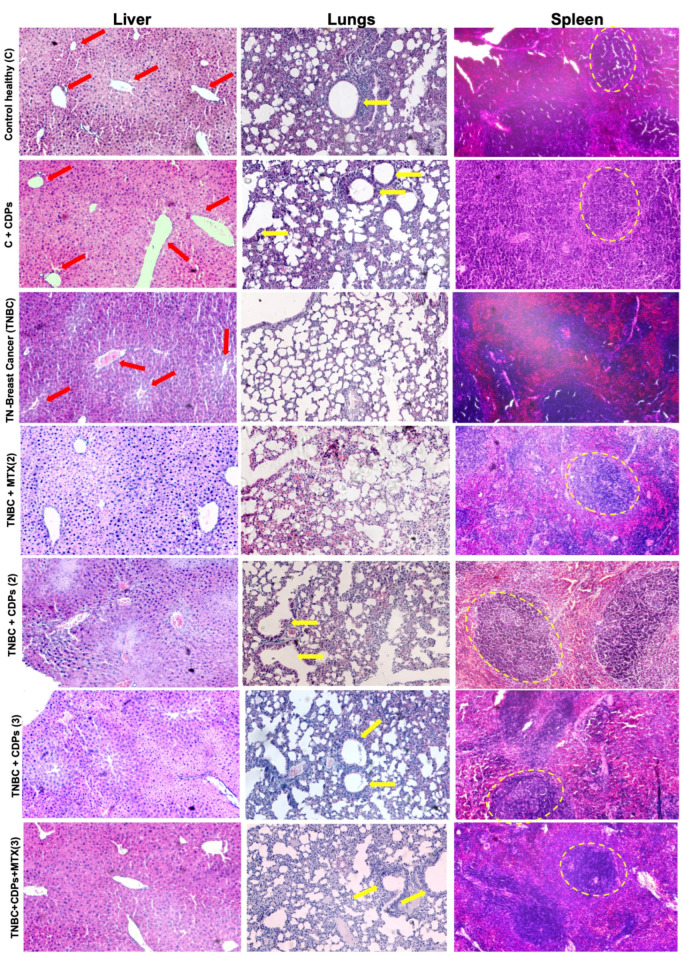
Histological features of the liver, lungs, and spleen of xenografted triple-negative breast cancer MDA-MB-231 cells in immunosuppressed mice treated with bacterial CDPs. Female BALB/c nu/nu mice (8 weeks old) were subjected to an anti-tumor study and analyzed at experimental endpoints of 35 or 70 days (n = 5 per group). Organ tissue sections were stained with hematoxylin and eosin (H&E) and examined by light microscopy. Representative images of tissues dissected from the animal groups are shown. The blood conductors (red arrows), alveolar structures (yellow arrows), and splenic cellular structures (dashed circles) are indicated in the histology images.

Histological examination of the liver sections showed reduced nuclear hyperchromasia in mice treated with CDPs, either alone or combined with methotrexate (MTX), compared to untreated TNBC mice, with no signs of hepatocellular or tissue hypertrophy (Figure 8). In lung sections, untreated TNBC mice exhibited significant disruption of normal alveolar architecture, while healthy control mice (C) and healthy mice treated with CDPs (C + CDPs) maintained well-preserved alveolar structures. Notably, in the tumor groups treated with CDPs and MTX, the alveolar structure was restored to resemble that of the healthy groups (see yellow arrows in Figure 8). Additionally, spleen histology revealed disintegration of structural features in the tumor-bearing animals (TNBC), which was reversed in the TNBC groups treated with CDPs, MTX, or both (see dashed circles in Figure 8).

### 2.7. Gene Expression in the Liver of Xenografted TNBC MDA-MB-231 Line Treated with Bacterial CDPs

**Figure 9:**
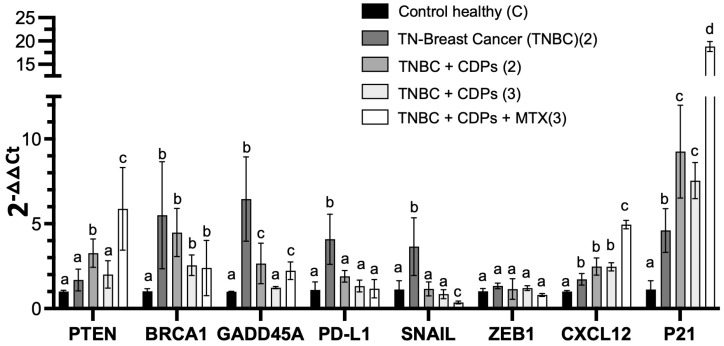
Hepatic gene expression analysis in an MDA-MB-231 xenograft mouse model of triple-negative breast cancer treated with bacterial CDPs. Relative expression levels of PTEN, BRCA1, GADD45A, PD-L1, SNAIL, ZEB1, CXCL12, and CDKN1A (p21) were determined in liver tissue by RT-qPCR. Gene expression was normalized to the housekeeping gene ACTB (β-actin), and relative expression levels were calculated using the 2^−ΔΔCt^ method. Experimental groups are defined as described in Figure 4. The bars indicate the means ± SE; n = 3 per group. Statistical analysis was performed using one-way ANOVA followed by Tukey’s post hoc test. SE values are shown as bars; within each gene, groups sharing the same lowercase letter are not significantly different from each other, whereas groups with different letters indicate statistically significant differences (*p* < 0.05).

The PI3K/Akt/mTOR and FOXO1 signaling pathways play crucial roles in fundamental cellular processes such as development, metabolism, and cell survival. Their dysregulation contributes to systemic changes associated with tumors. Based on our in vitro and in vivo results showing that CDP treatment modulates Akt, Gab1, and FOXO1 in the orthotopic TNBC xenograft mouse model, we aimed to further investigate the molecular mechanism behind CDP-mediated tumor inhibition. To do this, we examined the regulation of genes involved in pathways like cell proliferation, metastasis, apoptosis, immune response, and drug resistance. Gene expression analysis was carried out using RT-qPCR on liver tissues from healthy mice, untreated TNBC mice, and TNBC mice treated with CDPs, methotrexate (MTX), or their combination (Figure 9). The tumor suppressor genes PTEN and CDKN1A (p21), along with the chemokine CXCL12, were significantly upregulated in the livers of TNBC-bearing mice compared to healthy controls. Notably, their expression was substantially higher with CDP treatment, especially in combination with MTX (Figure 9). Conversely, ZEB1 showed no significant differences among the experimental groups. Interestingly, genes such as BRCA1, GADD45A, PD-L1, and SNAIL were markedly elevated in the livers of TNBC mice compared to healthy controls, but their expression decreased notably with CDP and MTX treatments, sometimes reaching levels similar to those in healthy mice (Figure 9).

## 3. Discussion

CDPs have become promising options for cancer treatment due to their stability, high selectivity, and ability to block key cancer signaling pathways [11]. Their cytotoxic and pro-apoptotic effects have been shown in multiple cancer cell lines [3,5,6,7,8]. Beyond their direct anti-proliferative activity, several diketopiperazines have been shown to interfere with mechanisms associated with tumor aggressiveness and therapeutic resistance. For example, fumitremorgin C analogs can overcome multidrug resistance by inhibiting the breast cancer resistance protein (BCRP), a key efflux transporter that limits intracellular drug accumulation. Similarly, the HLY838 diketopiperazine functions as an O-GlcNAc transferase (OGT) inhibitor, enhancing the anti-tumor effects of CDK9 inhibitors by downregulating c-Myc and E2F1 [12].

Additionally, Verticillin A inhibits c-Met phosphorylation and the downstream Ras/Raf/MEK/ERK signaling pathway, resulting in reduced metastatic potential in colon cancer models [13]. Together, these studies highlight the potential of CDPs and related diketopiperazines to serve as multitarget anticancer and anti-metastatic agents. Consistent with this, earlier research in mouse melanoma showed that CDPs from Pseudomonas aeruginosa significantly decreased key EMT markers, including MMP-1, E-cadherin, N-cadherin, HIF-1α, Vimentin, and CK-1, supporting their ability to inhibit tumor cell migration and invasion [6,10]. These observations support investigating the anti-invasive and metastasis-related effects of bacterial CDPs in aggressive breast cancer subtypes like triple-negative breast cancer.

In this study, functional assays further confirm the ability of bacterial CDPs to suppress invasion and metastasis-associated behaviors in the TNBC model. Wound-healing assays demonstrated that CDPs inhibit the migratory capacity of MDA-MB-231 cells more effectively than methotrexate (MTX) (Figure 1a). Similarly, transwell invasion assays show that CDPs significantly reduce the invasive ability of MDA-MB-231 cells under both monoculture and macrophage co-culture conditions compared to MTX, with their combination being even more effective. Tumor-associated macrophages (TAMs) play a key role in tumor progression by secreting chemokines and growth factors that enhance cancer cell proliferation, migration, and invasiveness [14]. Our data indicate that CDPs interfere with TAM–cancer cell interactions in the MDA-MB-231 cell line, highlighting their dual role in promoting apoptosis and reducing metastasis-related processes. This supports their function as inhibitors of tumor cell migration and invasiveness. The data are particularly relevant because the interaction between cancer cells and macrophages, along with the release of chemokines, enhances malignancy and the invasive capacity of cancer cells. The greatest inhibition observed with the combination of CDPs and MTX (up to 90% reduction in invasiveness) suggests a synergistic effect between these agents.

Three-dimensional spheroid models further demonstrated the anti-metastatic and anti-tumor effects of CDPs. Spheroid cultures are enriched with cancer stem cell (CSC)-like populations that exhibit increased self-renewal and resistance to therapy [14]. Therefore, the spheroid structure in MDA-MB-231 cells promotes resistance to cytotoxic compounds. Our results showed that CDPs significantly reduced spheroid size and number, lowered cell viability, and increased apoptosis in MDA-MB-231 spheroids compared to MTX-treated cultures. The effects were more pronounced when using the CDPs + MTX combination or cyclodextrins (Figure 1c–e). Additionally, the spheroid integration assay, which mimics three-dimensional tumor growth and plasticity, supports the ability of CDPs to disrupt key stages of tumor progression and stemness-related properties. These findings highlight the potential of CDPs to overcome CSC-associated resistance mechanisms in aggressive breast cancer models.

Mechanistically, our results further support previous findings that indicate CDPs act through the PI3K/Akt/mTOR pathway [3,5,6,10]. In MDA-MB-231 cells, CDPs significantly decreased phosphorylation of Akt, mTOR, and S6K (Figure 2), as well as reducing levels of Vimentin and Gab1—two proteins closely linked to EMT-related and metastasis-associated signaling [15]. Thus, CDPs effectively target the PI3K/Akt/mTOR signaling pathway, a key regulator of cell survival, proliferation, and growth. This aligns with the observed suppression of p-S6K phosphorylation and the decrease in Vimentin and p-Gab1 expression. Vimentin is an essential marker of EMT, a process crucial for invasiveness and metastasis. Therefore, the downregulation of Vimentin and inhibition of the PI3K/Akt/mTOR pathway are primary molecular mechanisms responsible for the reduced migration and invasiveness observed in vitro in MDA-MB-231 cells.

Beyond protein-coding genes, growing evidence highlights the role of non-coding RNAs (ncRNAs), including lncRNAs and miRNAs, as key regulators of EMT, metastasis, and therapeutic resistance in breast cancer. Our previous transcriptomic analysis of bacterial cyclodipeptides (CDPs) in the HeLa cell line revealed modulation of transcription factors and regulatory genes involved in epigenetic control and the regulation of miRNAs, circRNAs, and lncRNAs, suggesting that ncRNA-mediated mechanisms may contribute to the anticancer effects of CDPs [9]. Although ncRNAs were not directly examined in this study, the observed suppression of the PI3K/Akt/mTOR/FOXO1 pathway and EMT-related markers aligns with signaling pathways known to be regulated by metastasis-associated lncRNAs. Notably, MALAT1 has been shown to promote EMT, activate PI3K/Akt/mTOR signaling, and inhibit FOXO1-mediated tumor suppressive functions in breast cancer [16]. In this context, the coordinated suppression of Akt phosphorylation, EMT markers, and FOXO1 inactivation after CDP treatment may indicate the involvement of molecular pathways controlled by MALAT1 and related ncRNAs.

Meanwhile, emerging therapies focus on targeting metastasis-related non-coding RNAs with advanced delivery systems. Notably, exosome-mediated siRNA delivery has been shown to inhibit postoperative breast cancer metastasis by disrupting pro-metastatic signaling and pre-metastatic niche formation in triple-negative breast cancer models [17]. Compared to earlier research, the current findings indicate that CDPs might have anti-metastatic effects by indirectly affecting signaling pathways often regulated by ncRNAs, such as the PI3K/Akt/mTOR/FOXO1 pathway, without requiring complex delivery methods.

In the orthotopic TNBC xenograft model, implanting MDA-MB-231 cells directly into the mammary gland of animals creates a tumor microenvironment that exhibits key features of aggressive breast cancer progression. Importantly, before assessing antitumor efficacy, histopathological evaluation of major organs combined with continuous monitoring of body weight showed no evidence of systemic toxicity related to CDP treatment, supporting a favorable in vivo safety profile (Figure 8 and Figure S1). Treatment with CDPs significantly reduced tumor volume and weight, especially when administered after the tumor was established, and many treated animals showed no detectable tumors at the end of the experiment (Figure 4). Besides suppressing primary tumor growth, CDP treatment also alleviated tumor-associated systemic changes. Untreated TNBC-bearing mice had increased weights of the lungs, kidneys, liver, and spleen, along with histological changes such as metastatic foci indicating organ damage caused by the tumor (Figure 4). Conversely, groups treated with CDPs and the combination of CDPs plus MTX showed normalization of organ weights and no visible metastatic foci. Histopathological analysis of primary tumors revealed that untreated TNBC displayed extensive hyperchromasia. In contrast, CDP-treated tumors showed less hyperchromasia, with areas of cell aggregation observed (Figure 6). These findings suggest that in tumors from animals treated with CDPs and MTX, cell proliferation is inhibited, as indicated by the size and weight of the tumors dissected from each group (Figure 4).

Metastatic progression in breast cancer patients is often linked to systemic changes, including increased levels of serum ALT, AST, gamma-glutamyl transferase (GGT), alkaline phosphatase (ALP), and LDH [18]. In our TNBC model, untreated mice showed significant increases in AST and LDH (Figure 5). Treatment with CDPs normalized these enzyme levels and reduced organ weight gains. Changes in leukocyte profiles, especially a higher neutrophil-to-lymphocyte ratio (NLR), are recognized markers of poor prognosis in breast cancer [19]. In our study, TNBC mice exhibited the highest NLR, which was reduced in the CDP-treated groups, approaching control levels (Figure 5c), indicating decreased tumor aggressiveness. Anemia, another common cancer complication, was also improved with CDP treatment. These findings suggest that CDPs mitigate tumor-related systemic and liver dysfunction, supporting a reduction in tumor aggressiveness and metastatic potential.

It is important to emphasize the safety profile and systemic benefits. CDP treatment did not change the activity of liver enzymes ALT, AST, and LDH in healthy control mice (C + CDPs). Conversely, CDPs reversed the significant increase in AST and LDH activity observed in the TNBC group. These results, along with the restoration of histological structures in the lung and spleen, show that CDPs not only target the tumor locally but also help normalize tumor-related systemic changes. Histological analysis revealed a clear reduction in vascular structures in the liver of the TNBC group. In contrast, structural damage was observed in the lung and spleen tissues, which was significantly reduced after CDP treatment (Figure 8). These findings further suggest that, in our model, metastasis of the MDA-MB-231 line occurred in the animals following implantation into target organs such as the liver, lungs, and spleen. The ability of CDPs to restore normal tissue architecture supports their role in reducing tumor-related organ dysfunction and decreasing metastatic risk.

At the molecular level, protein and gene expression analyses revealed coordinated modulation of signaling pathways involved in metastasis, immune evasion, and DNA damage response. To further explore tumor-associated signaling in TNBC, we assess metastasis in the tumor of the xenografted TNBC model. Molecular analyses also showed reduced p-Akt levels in tumors from CDP-treated animals, along with decreased total Akt expression (Figure 7), indicating suppression of the PI3K/Akt/mTOR signaling pathway. FOXO1, a forkhead transcription factor that regulates apoptosis, autophagy, and cell cycle arrest, is negatively regulated by Akt-mediated phosphorylation [20]. In our study, CDP-treated animals showed decreased FOXO1 phosphorylation. Although total FOXO1 protein levels were reduced, the decrease in FOXO1 phosphorylation corresponds with increased FOXO1 activity, allowing its nuclear functions and the activation of growth-suppressive and stress-response pathways [21]. This activation aligns with the induction of apoptotic pathways. These findings match our previous transcriptomic data in HeLa cells, showing CDP-mediated upregulation of FOXO1-related genes like GADD45A and SGK1 [9].

Gab1, an adaptor protein that mediates signals for proliferation, angiogenesis, and invasion [22], was overexpressed and phosphorylated in TNBC tumors but was downregulated by CDPs, especially when combined with MTX (Figure 7). This indicates that inhibiting Gab1 is a key mechanism behind the suppression of invasion- and metastasis-related processes caused by CDPs. Protein analysis in the xenografted tumors confirmed the inhibition of the Akt pathway. Consistent with the in vitro results, the expression of phosphorylated Akt protein was reduced in the CDP-treated groups, as Gab1, p-Gab1, FOXO1, and its phosphorylated form p-FOXO1 were markedly decreased in tumors from mice treated with CDPs and MTX.

The study of gene expression in the liver provided crucial data on tumor-related systemic changes influenced by the CDPs. To further explore the metastasis-related signaling pathways targeted by the CDPs, we analyzed the hepatic expression of gene markers associated with multiple pathways involved in tumor progression, immune regulation, and cellular plasticity (Figure 9). RT-qPCR analysis showed that CDP treatment significantly reversed the overexpression of key genes seen in the TNBC group, including SNAIL, which is linked to promoting EMT, invasiveness, metastasis, and drug resistance. Among immune checkpoint components, such as PD-L1, which contributes to immune evasion, overexpression was observed in the TNBC group and returned to normal levels after CDP treatment, approaching those seen in healthy controls. Regarding genes involved in DNA damage/repair and the cell cycle—such as BRCA1 and GADD45A, which exhibited a marked increase in the TNBC group consistent with tumor-induced systemic stress and genomic instability—these were normalized by CDP treatment. Conversely, genes like PTEN, CXCL12, and CDKN1A (p21) were overexpressed in the livers of animals treated with both CDPs and MTX compared to untreated healthy mice, indicating activation of compensatory tumor-suppressive and cell cycle–regulatory mechanisms related to EMT, invasiveness, metastasis, and drug resistance, reflecting an adaptive hepatic response aimed at reinforcing immune regulation, growth suppression, and homeostasis despite tumor burden. Overall, these findings suggest that bacterial CDPs in the TNBC model modulate tumor-associated systemic gene expression programs related to DNA damage responses, immune regulation, and cellular stress, thereby affecting molecular mechanisms connected to tumor aggressiveness and metastatic progression. This modulation targets both tumor cell–intrinsic signaling pathways and systemic responses linked to the tumor. The inhibition of Akt/mTOR/S6K signaling, suppression of EMT markers, modulation of immune-related genes, and the favorable safety profile collectively position CDPs as promising therapeutic agents. Moreover, the enhanced efficacy observed when combining CDPs with MTX supports the potential of combination strategies to improve treatment outcomes in aggressive breast cancer subtypes.

In summary, in the TNBC mouse model, CDP administration significantly reversed primary tumor growth in the mammary glands, with greater effectiveness when combined with MTX. Beyond local tumor control, CDP treatment reduced systemic changes associated with tumors in distant organs, such as the liver, which are commonly involved in metastatic progression. The treatment with CDPs reduced metastatic foci in organs such as the liver. At the molecular level, CDPs modulated the expression of genes linked to tumor aggressiveness, immune regulation, and stress responses, such as SNAIL, GADD45A, and PD-L1, while increasing the expression of growth-suppressing and homeostatic regulators, including PTEN, CXCL12, and CDKN1A (p21). The data provide new insights into the molecular mechanisms behind the anti-metastatic and anti-invasive effects of CDPs, showing that these effects are connected to genes involved in EMT, invasiveness, and metastasis. This indicates that the signal transduction mechanism involves inhibition of phosphorylation in the Akt/mTOR/S6K pathway and of metastasis markers, such as Vimentin, Gab1, and FOXO1. The findings suggest that combining CDPs with MTX enhances the anti-metastatic effect in a TNBC xenograft model using the MDA-MB-231 line, indicating that bacterial CDPs could be a promising therapeutic agent. In conclusion, bacterial cyclic peptides represent a promising multi-target therapy for TNBC, showing greater efficacy than MTX in vivo. Their mechanism involves blocking the PI3K/Akt/mTOR/FOXO1 pathway and suppressing EMT, resulting in strong anti-tumor and anti-metastatic effects with a good safety profile.

## 4. Materials and Methods

### 4.1. Chemicals, Reagents, and Cell Culture

The chemicals and reagents used include Dulbecco’s Modified Eagle Medium (DMEM; Sigma-Aldrich, St. Louis, MO, USA), fetal bovine serum (FBS; Gibco Life Technologies, Grand Island, NY, USA), and trypsin solution (Sigma-Aldrich). A mixture mainly composed of cyclo(L-Pro-L-Tyr), cyclo(L-Pro-L-Val), cyclo(L-Pro-L-Leu), and cyclo(L-Pro-L-Phe) is isolated from the cell-free supernatant of *Pseudomonas aeruginosa* PAO1 bacteria, with a purity of over 95% [23,24]. This mixture is dissolved in a DMSO-water ratio of 1:3 to prepare stock solutions (100 mg/mL). The MDA-MB-231 breast tumor cell line was obtained from invasive ductal carcinoma (ATCC, Manassas, VA, USA) and cultured in complete media [DMEM supplemented with 10% (*v*/*v*) FBS, 100 units/mL penicillin, 40 µg/mL streptomycin, and 1 µg/mL amphotericin B (Sigma-Aldrich)], with 1.6 g/L glucose added. Cell culture media were changed twice a week, with cells incubated at 37 °C, 80% humidity, and 5% CO_2_. Cells were then trypsinized and counted using a hemocytometer. All cell cultures and procedures were performed in class II biological safety cabinets.

### 4.2. Cell Viability and Apoptosis Determination

Cell viability was determined with the MTT method. Briefly, cell cultures were grown in 96-well flat-bottomed plates in DMEM medium containing FBS for 24 h, then incubated with CDPs for 24 h at 37 °C with 5% CO_2_. MTT (50 mg/mL) in PBS was added to each well, and the mixture was incubated for 4 h at 37 °C. Finally, 100 μL of 2-propanol/1M HCl (19:1, *v*/*v*) was added to dissolve the formazan crystals, and the absorbance was measured at 595 nm using a microplate reader (BioTek Instruments, Winooski, VT, USA).

Necrosis and apoptosis were evaluated in cell cultures incubated in DMEM medium with FBS for 4 h with CDP treatment. Following incubation, cells were collected by centrifugation at 2000× *g* for 10 min. The pellet was suspended in 20 μL and incubated with annexin V and propidium iodide (PI) (Molecular Probes, Invitrogen, Life Technologies, Carlsbad, CA, USA). Fluorescence was immediately quantified by FACS using an Accuri-C6 Flow Cytometer (BD Biosciences, San Jose, CA, USA). At least 20,000 cellular events were used for calculations.

### 4.3. Wound Closure Migration Assay

The MDA-MB-231 cell line was grown to 95% confluence, and three wounds were created per plate using a sterile pipette tip. All assays were performed in triplicate. After wounding, plates were washed twice with phosphate-buffered saline (PBS) and replenished with fresh complete medium. Cells were then treated with CDPs (0.01 mg/mL), methotrexate (MTX; 0.05 mg/mL), or their combination for 48 h. Representative images were taken every 24 h, and wound dimensions were quantified using ImageJ 1.53k software (NIH, Bethesda, MA, USA).

### 4.4. Invasion Assay

The invasive capacity of the MDA-MB-231 cell line was evaluated using a transwell chamber pre-coated with Matrigel (Corning Life Sciences, Union City, CA, USA). Cells were seeded in the upper chamber in complete DMEM medium. For co-culture experiments, RAW 264.7 macrophages were seeded in the lower chamber containing complete DMEM medium. Cells were treated with CDPs (0.01 mg/mL) or methotrexate (MTX; 0.05 mg/mL) incubated by 24 h at 37 °C with 5% CO_2_. Inserts were then briefly washed with PBS, and cells that had invaded through the Matrigel and adhered to the lower surface of the membrane were fixed with 70% ethanol for 15 min at room temperature. Fixed cells were stained with 0.2% (w/v) crystal violet for 5 min, washed thoroughly with PBS to remove excess dye, and air-dried. Invaded cells were visualized by optical microscopy [25].

### 4.5. Multicellular Spheroids

MDA-MB-231 cell line (2 x10^5^ cells) was cultured under non-adherent conditions using 0.6% agarose in complete DMEM medium to generate multicellular spheroids. Cultures were maintained for 14 days with periodic medium replacement until the spheroids reached a diameter of 40–50 µm [14]. Spheroids were then treated with CDPs (0.1 mg/mL) or methotrexate (MTX; 0.05 mg/mL) and incubated for 4 h, a time point selected to correspond with the determination of lethal (LD_50_) and apoptotic effective (ED_50_) doses. Following treatment, spheroids were visualized by optical microscopy, and cell viability was assessed using the MTT assay.

### 4.6. Western Blot Assays

Confluent MDA-MB-231 cells were treated with CDPs (0.1 mg/mL) and incubated for 15 min, 1 h, and 4 h. Cells were then harvested by trypsinization, washed twice with PBS, and centrifuged at 5000× *g* for 10 min at 4 °C. Cells pellets or tumor tissue were resuspended in RIPA lysis buffer, and lysed by three cycles of low- intensity sonication (20 kHz, 5 W, 30 s each) at 4 °C using an Hielscher LS24 ultrasonic processor. The protein extract free of cellular debris was obtained by centrifugation at 7500× *g* for 15 min, and the protein concentration was determined by the Bradford method (BioRad, Hercules, CA, USA). Equal amounts of protein were separated by 10% SDS-PAGE and transferred onto PVDF membranes (Millipore, Billerica, MA, USA) using a Bio-Rad transfer system at 15 volts for 45 min. Membranes were blocked with TBS-T (Tris-HCL 10 mM; NaCl 0.9%; tween-20 0.1%, dry milk 5%, pH 7.8), washed three times for 6 min each, and incubated overnight at 4 °C with primary antibodies diluted in TBS-T (1:5000). The following primary antibodies were used: anti-Akt (cat. 2938), anti-p-Akt (cat. 4060), anti-mTOR (cat. 2983), anti-p-mTOR (cat. 5536), anti-S6K (cat. 2708), anti-p-S6K (cat. 9234), anti-Vimentin (cat. 3932); anti-Gab1 (cat. 3232), anti-p-Gab1 (cat. 3234), anti-FOXO1 (cat. 2880), anti-p-FOXO1 (cat. 9464), and anti-β-actin (cat. 4967) (Cell Signaling Technology, Boston, MA, USA and Santa Cruz Biotechnology, Santa Cruz, CA, USA). After three washes with TBS-T (6 min each), membranes were incubated with HRP-conjugated secondary antibody (BioRad) diluted 1:10,000 in TBS-T for 2 h at room temperature. Immunoreactive bands were detected using SuperSignal™ West Pico chemiluminescent substrate (Pierce; Thermo Fisher Scientific, Waltham, MA, USA) and visualized with a ChemiDoc™ MP imaging system (Bio-Rad). Assays were conducted at least three times, and representative images are shown. ImageJ (NIH) was used to quantify image band intensities.

### 4.7. Xenografted Metastatic Breast Cancer Model

Immunosuppressed female BALB/c nu/nu mice (8 weeks old) were acclimated for 15 days in a pathogen-free environment with a standard diet and water provided *ad libitum*. All animal procedures were approved by the Institutional Animal Care and Use Committee (IACUC) from the Universidad Michoacana de San Nicolás de Hidalgo (IIIQB-UMSNH-IACUC-2022-35). Experiments complied with standard guidelines for the welfare of animals following the Institutional Committee and recommendations of the Mexican Official Regulations for the Use and Care of Animals (NOM 062-ZOO-1999; Ministry of Agriculture, Mexico).

Once the acclimation period concluded with 100% of animals survival, mice were orthotopically implanted with 1 × 10^5^ MDA-MB-231 metastatic breast cancer cells suspended in Matrigel and injected into the mammary fat pad adjacent to the right axillary region. Animals were anesthetized by intraperitoneal administration of ketamine (80 mg/kg) and xylazine (10–15 mg/kg). Body weight was monitored throughout the experiment, and tumor dimensions were measured with a caliper. Tumor volume (TV) was calculated using the formula TV = (0.4) (ab^2^), where “a” represents the longest tumor diameter and “*b*” the shortest diameter. In addition, a pilot study was carried out to select the doses of CDPs to be tested in the TNBC mice model; thus, we decided to use 0.25 mg/kg of mouse body weight. Treatments were administered intraperitoneally and consisted of CDPs (0.25 mg/kg), methotrexate (MTX; 0.05 mg/kg), or a combination of CDPs and MTX (0.25 mg/kg CDPs + 0.05 mg/kg MTX). The CDP treatments involved three rounds of administration, each consisting of three doses given every 3 days (with a 2-day rest between doses), and a week of rest between rounds, totaling 9 doses per mouse. The animal groups, each consisting of five mice (n = 5) as follows (Figure 3): healthy mice without treatment (C)—procedure 1; healthy mice treated with CDPs (C + CDPs)—procedure 1; mice with TNBC tumors without treatment (TNBC)—procedure 2; mice with tumors treated with CDPs starting from cell inoculation in early stage (TNBC + CDPs (2))—procedure 2; mice with tumors treated with CDPs at the 35th day post-inoculation in advanced stage (TNBC + CDPs (3))—procedure 3; mice with advanced-stage tumors treated with MTX (TNBC + MTX (3))—procedure 3; and mice with tumors treated with a combination of CDPs and MTX (TNBC + CDPs + MTX (3))—procedure 3.

### 4.8. Animal Euthanasia and Biological Sample Collection

All mice were euthanized by intraperitoneal administration of a lethal dose of sodium pentobarbital (100–150 mg/Kg body weight), as recommended by IIQB/UMSNH/-IACUC and NOM 062-ZOO-1999. Following confirmation of death, tumors were excised, and whole blood was collected by cardiac puncture into microtubes containing EDTA as an anticoagulant. Major organs were subsequently harvested and weighed.

Blood hemoglobin was determined using heparinized capillary tubes by centrifugation at 3500 rpm for 5 min to separate red blood cells from plasma. Subsequently, serum biochemical parameters, including aspartate aminotransferase (AST), alanine aminotransferase (ALT), and lactate dehydrogenase (LDH), were quantified using a Fuji Dry-Chem NX700 (Fujifilm, Santa Ana, CA, USA).

### 4.9. Histological Analysis of Tissues

After euthanasia, tumors, liver, lungs, and spleen were excised and processed for histological evaluation. Tissues were fixed in 4% paraformaldehyde and remained until dehydration. The samples were subjected to a 4 h dehydration process at varying ethanol and xylene concentrations, then embedded in paraffin. Paraffin-embedded tissues were sectioned at a thickness of 4 µm, mounted on glass slides, and stained with hematoxylin and eosin (H&E). Photographs were taken under an optical microscope and recorded using an Accu-Scope EXC-120 LED microscope camera at magnifications of 4×, 10×, and 40×.

### 4.10. RNA Extraction and RT-qPCR

Total RNA from liver samples of each animal group was pooled, homogenized using a tissue homogenizer (Biospec Products, Bartlesville, OK, USA), and extracted using TRIzol reagent (Invitrogen; Thermo Fisher Scientific) according to the manufacturer’s protocol. The RNA concentration and quality were assessed using a spectrophotometer (NanoDrop 2000; Thermo Fisher Scientific). According to the manufacturer’s instructions, complementary DNA (cDNA) was synthesized using a QuantiNova Reverse Transcription Kit (Bio-Rad). Quantitative PCR (qPCR) was performed using the Quantinova SYBR Green PCR Kit (Bio-Rad) in a reaction volume of 10 µL. The thermocycling conditions were set as follows: initial denaturation at 95 °C for 10 min, followed by 40 cycles of denaturation at 95 °C for 5 s and annealing/extension at 60 °C for 30 s and extension to 68 °C for 30 s using a thermocycler (QuantStudio 3; Applied Biosystems by Thermo Fisher Scientific). Relative gene expression levels was calculated using the comparative 2^−ΔΔCt^ method, normalizing to the internal reference gene *ACTB* (β-actin). All experiments were conducted in triplicate. The sequences of the gene-specific primers are provided in Table S1.

### 4.11. Statistical Analysis

All quantitative data are presented as mean ± standard error (SE). Statistical analyses were performed using one-way analysis of variance (ANOVA) when comparing more than two experimental groups. When ANOVA indicated significant differences, Tukey’s post hoc multiple comparison test was applied to identify statistically significant pairwise differences between groups. A *p*-value < 0.05 was considered statistically significant. All analyses were performed using GraphPad Prism version 6.0.

## Data Availability

The data supporting the findings of this study are available from the corresponding author upon request.

## Supplementary Materials

The following supporting information can be downloaded at: https://www.mdpi.com/article/10.3390/molecules31030543/s1, Figure S1: The mouse body weight was monitored during the therapeutic experimental outline study; Table S1: Oligonucleotides used in this study.
