# Supplementary material for: Bacterial Cyclodipeptides Inhibit Invasiveness and Metastasis Progression in the Triple-Negative Breast Cancer MDA-MB-231 Mouse Model
Source: Molecules. 2026 Feb 4;31(3):543. doi: 10.3390/molecules31030543 (PMC12899019; doi:10.3390/molecules31030543)
Supplement: Supplementary file 1 [file molecules-31-00543-s001.zip › molecules-4118657-supplementary/Figure S1.pdf]

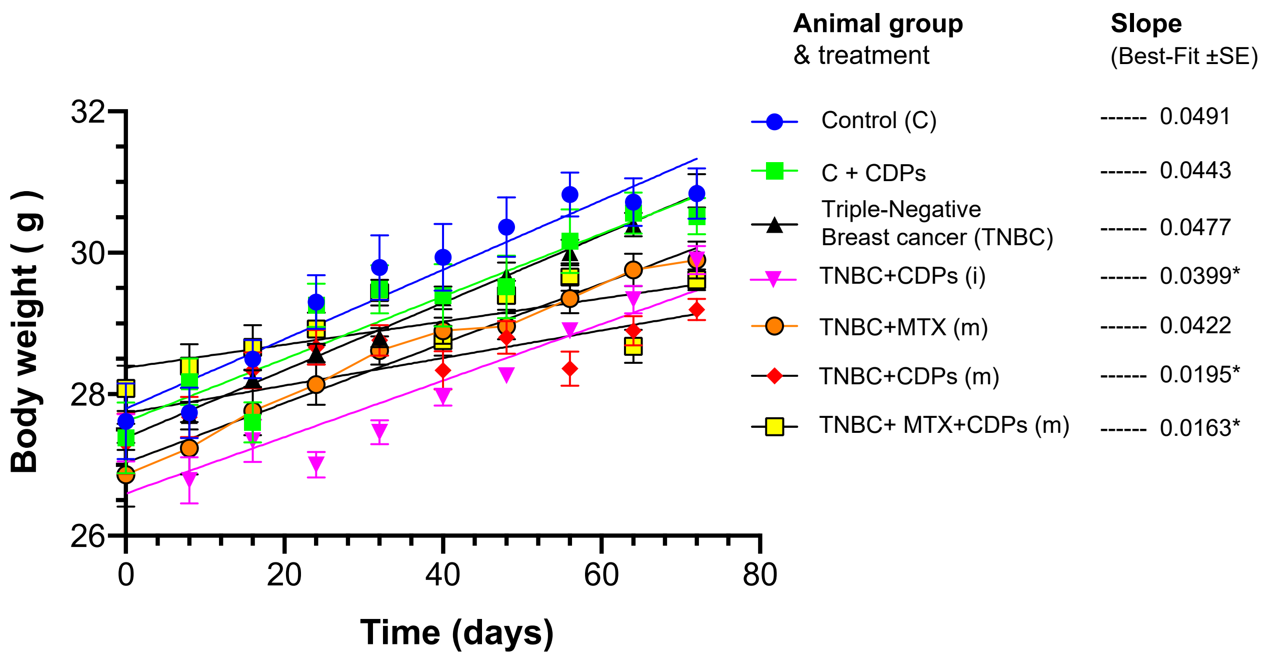

Figure S1. The mouse body weight was monitored during the therapeutic experimental outline study. The slope of weight gain values is shown in all the mice groups (right). Statistical analysis was performed using t-student; values are displayed, and significant differences are indicated with asterisks (p <0.05), n=5 per group. Mice groups: Control (C), healthy animals control without treatment; C+CDPs, healthy animals administered with CDPs; TNBC, animals that developed breast cancer without treatment; TNBC+CDPs (i), TNBC animals administered with CDPs as the procedure 1; TNBC+CDPs (m), TNBC animals administered with CDPs as procedure 2; TNBC+MTX (m), TNBC animals administered with methotrexate (MTX) as procedure 2; TNBC+CDPs+MTX (m), TNBC animals co-administered with CDPs + methotrexate as procedure 2.
