# Supplementary material for: Bacterial Cyclodipeptides Inhibit Invasiveness and Metastasis Progression in the Triple-Negative Breast Cancer MDA-MB-231 Mouse Model
Source: Molecules. 2026 Feb 4;31(3):543. doi: 10.3390/molecules31030543 (PMC12899019; doi:10.3390/molecules31030543)
Supplement: Supplementary file 1 [file molecules-31-00543-s001.zip › molecules-4118657-supplementary/Table S1.pdf]

Table S1. Oligonucleotides used in this study.

Gene	Forward	Reverse	Reference
PTEN FW	TGAGTTCCCTCAGCCATTGCCT	GAGGTTTCCTCTGGTCCTGGTA	Hakeem et al., 2024
BRCA1	CGAGGAAATGGCAACTTGCCTAG	TCACTCTGCGAGCAGTCTTCAG	Nie et al., 2024
GADD45A	CCTGGAGGAAGTGCTCAGCAAG	GTCGTCTTCGTCAGCAGCCAG	Tian et al., 2016
PD-L1	TGCGGACTACAAGCGAATCACG	CTCAGCTTCTGGATAACCCTCG	Yang et al., 2025
p21	TCGCTGTCTTGCACTCTGGTGT	CCAATCTGCGCTTGGAGTGATAG	Cao et al., 2021
SNAI1	TGCCCTCAAGATGCACATCCGA	GGGACAGGAGAAGGGCTTCTC	Zhong et al., 2023
ZEB1	GGCATACACCTACTCAACTACGG	TGGGCGGTGTAGAATCAGAGTC	Zhong et al., 2023
CXCL12	TCAGCCTGAGCTACAGATGC	CTTTAGCTTCGGGTCAATGC	Li et al., 2011
ACTB	CATTGCTGACAGGATGCAGAAGG	TGCTGGAAGGTGGACAGTGAGG	Tian et al., 2016

References

1. Cao, Y., Wu, H., Cao, X., Yue, K., Han, W., Cao, Z., Zhang, Y., Gao, X., Luo, C., Jiang, X., Han, H., & Zheng, M. (2021). Transmembrane Protein Ttyh1 Maintains the Quiescence of Neural Stem Cells Through Ca2+/NFATc3 Signaling. Frontiers in Cell and Developmental Biology, 9. https://doi.org/10.3389/fcell.2021.779373
2. Hakeem, A. N., El-Kersh, D. M., Hammam, O., Elhosseiny, A., Zaki, A., Kamel, K., Yasser, L., Barsom, M., Ahmed, M., Gamal, M., & Attia, Y. M. (2024). Piperine enhances doxorubicin sensitivity in triple-negative breast cancer by targeting the PI3K/Akt/mTOR pathway and cancer stem cells. Scientific Reports, 14(1), 18181. https://doi.org/10.1038/s41598-024-65508-0
3. Li, T., Li, H., Wang, Y., Harvard, C., Tan, J.-L., Au, A., Xu, Z., Jablons, D. M., & You, L. (2011). The expression of CXCR4, CXCL12 and CXCR7 in malignant pleural mesothelioma. The Journal of Pathology, 223(4), 519-530. https://doi.org/10.1002/path.2829
4. Nie, H., Saini, P., Miyamoto, T., Liao, L., Zielinski, R. J., Liu, H., Zhou, W., Wang, C., Murphy, B., Towers, M., Yang, T., Qi, Y., Kannan, T., Kossenkov, A., Tateno, H., Claiborne, D. T., Zhang, N., Abdel-Mohsen, M., & Zhang, R. (2024). Targeting branched N-glycans and fucosylation sensitizes ovarian tumors to immune checkpoint blockade. Nature Communications, 15(1), 2853. https://doi.org/10.1038/s41467-024-47069-y

5. Tian, Y., Wang, J., Wang, W., Ding, Y., Sun, Z., Zhang, Q., Wang, Y., Xie, H., Yan, S., & Zheng, S. (2016). Mesenchymal stem cells improve mouse non-heart-beating liver graft survival by inhibiting Kupffer cell apoptosis via TLR4-ERK1/2-Fas/FasL-caspase3 pathway regulation. Stem Cell Research & Therapy, 7(1), 157. https://doi.org/10.1186/s13287-016-0416-y
6. Yang, X., Chen, W., Sun, H., Chen, W., Xu, W., He, C., Liu, Y., Kuang, Y., Ma, Y., Zhong, B., Li, C., Li, G., Du, Q., & He, S. (2025). Emodin promotes GSK-3β-mediated PD-L1 proteasomal degradation and enhances anti-tumor immunity in hepatocellular carcinoma. Chinese Medicine, 20(1), 126. https://doi.org/10.1186/s13020-025-01146-6
7. Zhong, G., Zhao, Q., Chen, Z., & Yao, T. (2023). TGF-β signaling promotes cervical cancer metastasis via CDR1as. Molecular Cancer, 22(1), 66. https://doi.org/10.1186/s12943-023-01743-9
